# Self-reported HPV vaccination status and HPV vaccine hesitancy in a nationally representative sample of emerging adults in Croatia

**DOI:** 10.3389/fpubh.2023.1182582

**Published:** 2023-11-06

**Authors:** Tatjana Nemeth Blažić, Ivana Božičević, Mirjana Lana Kosanović Ličina, Aleksandar Štulhofer, Iskra Alexandra Nola

**Affiliations:** ^1^Department for HIV, Sexual and Blood Transmitted Diseases, Croatian Institute of Public Health, Zagreb, Croatia; ^2^Andrija Stampar School of Public Health, School of Medicine, University of Zagreb, Zagreb, Croatia; ^3^Department of Epidemiology of Infectious Diseases, Andrija Stampar Teaching Institute of Public Health, Zagreb, Croatia; ^4^Department of Sociology, Faculty of Humanities and Social Sciences, University of Zagreb, Zagreb, Croatia

**Keywords:** HPV, HPV vaccination, self-reported HPV vaccination status, vaccine hesitancy, emerging adults, Croatia

## Abstract

**Introduction:**

The aim of this study was to determine self-reported human papillomavirus (HPV) vaccination status among emerging adults in Croatia, intention to get the HPV vaccine among unvaccinated individuals and correlates of HPV vaccine hesitancy.

**Methods:**

Data were collected via a cross-sectional survey based on a probabilistic national sample. The sample included 1,197 individuals aged 18–25 years (50.6% were women) who were recruited from November 2021 to February 2022 as part of the commercial online panel. Analyses included 981 participants who correctly answered two “attention trap” questions using descriptive statistics and multivariate analyses. The data were *post-hoc* weighted for gender and age and adjusted for clustering effect. To adjust standard errors for the sampling design, multivariate analyses were carried out using the complex samples module in the IBM SPSS 27 statistical software package.

**Results:**

Overall, 18.3% of participants (25.0% of women and 11.7% of men) reported that they were HPV vaccinated, while 21.9% did not know their HPV vaccination status. Of those vaccinated, 65.6% were women. The odds of being HPV-vaccinated were significantly higher among female participants. Among the unvaccinated, 35.4% expressed a willingness to get the vaccine. The odds of vaccination hesitancy were significantly lower among women, participants who reported a higher perceived risk of STIs, those who recognized that HPV could result in cervical cancer, and significantly higher among those who were more religious.

**Conclusion:**

Our findings suggest a need to increase HPV vaccination uptake in Croatia through raising awareness about HPV vaccine effectiveness and also through the implementation of strategies to make vaccination more available.

## Introduction

Vaccination against human papillomavirus (HPV) is an important biomedical intervention for the prevention of HPV infection and associated cancers in women and men (1). HPV vaccination against the most common low- and high-risk HPV subtypes has been introduced in publicly funded HPV national immunization programs in many European countries since 2006 (2). Though there is strong evidence of HPV vaccination effectiveness, HPV vaccine hesitancy and low HPV vaccination coverage rates (HPV-VCR) remain a challenge (1).

In Croatia, HPV vaccination became available at the national level in 2007 for girls and women, aged 9–26 years, as an out-of-pocket payment. Some counties provided free HPV vaccinations for 13-year-old girls, accompanied by public campaigns (3, 4). Since 2016, HPV vaccination has been included in the national immunization program, covered by health insurance, and recommended for girls and boys aged 14–15 years. Starting in 2019, a catch-up campaign was introduced for all young, unvaccinated people aged up to 25 years. Since 2023, HPV vaccination has been recommended and offered on a voluntary basis to all children aged 10/11–14/15 years. Specialists in school and adolescent medicine actively recommend HPV vaccination to schoolchildren during their routine preventive activities. The catch-up free vaccination for individuals up to 25 years of age is available on request (3, 4). According to the unpublished implementation data of the Service for School Medicine of the Croatian Institute of Public Health, in the school year 2019/2020, 38% of the first-grade high school female students (aged 15–16 years) and 24% of the male students had been fully vaccinated.

HPV-VCRs in Croatia are not regularly monitored, unlike mandatory vaccinations that are included in the national immunization program during childhood and adolescence, where VCRs are set at 95% for all vaccinated cohorts. Therefore, population-based studies done at the national level can give much-needed information about the coverage of vaccination against HPV in the absence of routinely collected data.

The aim of this study was to determine self-reported HPV vaccination status against HPV in a population-based sample of emerging Croatian adults (young people aged 18–25 years), intention to get the vaccine among unvaccinated individuals and correlates of HPV vaccination and vaccine hesitancy.

## Methods

### Participants

The sample included 1,197 emerging adults aged 18–25 years (*M*age = 21.7, *SD* = 2.21; 50.6% women). Two-stage stratified sampling by region (*n* = 6) and settlement size (*n* = 4) was used to randomly draw participants in the selected age range from a commercial panel database (*n* = 5,000) based on quotas for age, gender, and education from 19 November 2021 to 11 January 2022. The database, maintained by a range of international professional research companies, consisted of participants who were recruited to join the panel while taking part in national, probability-based, face-to-face, or phone surveys and those who joined through the agency's web portal. Panel membership is incentivized.

Considering the COVID-19 restrictions, a commercial panel was the most feasible and efficient recruitment method. The study response rate was 29%, and the questionnaire completion rate was 83%. After excluding participants who failed to correctly answer one or both “attention trap” questions, the sample was reduced to 981 participants (52.1% women). The basic sociodemographic characteristics of this analytical sample (*n* = 981) are presented in the Supplementary Table 1.

To be broadly representative of the respective population, the sample was weighted for gender and age based on the most recent (2021) census data by the Croatian Bureau of Statistics and adjusted for the clustering effect. The basic sociodemographic characteristics of this analytical sample (*n* = 976) are presented in Table 1.

**Table 1:** Basic sociodemographic characteristics of the sample by gender (weighted data; *n* = 976)^*^.

	**Women**	**Men**	**Total**
	***n*** **(%)**	***n*** **(%)**	***n*** **(%)**
**Mother's education**			
Primary	53 (11.3)	36 (7.3)	90 (9.3)
Secondary	320 (65.9)	306 (62.3)	625 (64.1)
Tertiary	107 (22.0)	148 (30.2)	255 (26.1)
Unknown	4 (0.8)	1 (0.2)	5 (0.5)
**Father's education**			
Primary	63 (13.0)	36 (7.3)	99 (10.1)
Secondary	324 (66.7)	308 (62.9)	632 (64.8)
Tertiary	92 (19.0)	145 (29.5)	237 (24.3)
Unknown	7 (1.4)	2 (0.4)	8 (0.9)
**Relationship status**			
Married	19 (3.9)	12 (2.5)	31 (3.2)
Cohabiting	72 (14.8)	58 (11.8)	130 (13.3)
In a relationship but not cohabiting	229 (47.2)	190 (38.8)	419 (43.0)
Single	164 (33.7)	225 (46.0)	389 (39.9)
Something else	2 (0.3)	4 (0.9)	6 (0.6)
**Settlement type**			
Rural	217 (44.8)	153 (31.3)	371 (38.0)
Urban	268 (55.2)	337 (68.7)	605 (62.0)
	***M*** **(*****SD*****)**	***M*** **(*****SD*****)**	***M*** **(*****SD*****)**
Age (years)	21.7 (2.20)	21.8 (2.22)	21.8 (2.21)
Education (years of formal education)	7.3 (2.06)	6.8 (2.19)	7.0 (2.14)
Socioeconomic standing (5-point Likert scale)^**^	3.1 (0.66)	3.3 (0.68)	3.2 (0.67)
Religious upbringing (7-point scale)^**^	4.7 (1.59)	4.5 (1.61)	4.5 (1.60)
Religiosity (freq. of church attendance)^**^	3.8 (1.51)	3.5 (1.55)	3.7 (1.53)

^*^Numbers do not always add up due to weighting and rounding up.

^**^Explained under the Section Measures.

### Procedures

The study was conducted as the third wave of a repeated cross-sectional national study on sexual and reproductive health in emerging adults (5). It entailed a behavioral and a biological part (testing for *Chlamydia trachomatis*, which will be reported on separately). Here, data from the behavioral part of the study are presented. Online recruitment was initiated with a letter announcing the study and its contents. To access the questionnaire, participants needed to provide informed consent. Following the completion of the questionnaire, we used incentives to increase the chance of a better response (increasing the response rate) and better data quality. Participants received a small token of appreciation for a completed questionnaire (5 EUR voucher). Participation in the biological part of the study was awarded with a 20-EUR voucher. All study procedures were approved by the Ethical Committees of Faculty of the Humanities and Social Science and the Croatian Institute of Public Health.

### Measures

Dependent variables. Within the questionnaire, the HPV vaccination status (customarily takes place at the age of 14–15 years) was checked (investigated). Individuals who were either unvaccinated or could not remember being vaccinated were asked if they would like to “get vaccinated against HPV” (“yes”; “no”).

Independent variables. Participant's education (measured in years of formal education), comparative socioeconomic standing (answers were recorded on a 5-point Likert-like scale ranging from 1 = “my household income is much lower compared to most other households to 5 = “my household income is much higher compared to most other households”), relationship status (married or cohabiting, in a relationship but not cohabiting, and single), and religious upbringing (a 7-point scale ranging from 1 = “I was not brought up in a religious spirit” to 7 = “I was brought up to strictly follow religious principles” was used to explore family religiosity) were asked. Religiosity was assessed using a standard indicator of the frequency of attending religious ceremonies (1 = not religious, 2 = never… to 7 = almost every day). The self-assessed personal risk of acquiring sexually transmitted infections (STI) (1 = “the risk is negligible” to 10 = “the risk is extremely high”) was also queried. Basic knowledge about the potential consequences of HPV infection was indicated by the following question: “Is the protracted infection with HPV the main cause of cervical cancer?” (“no”; “yes”; and “I do not know”). Finally, settlement type (rural vs. urban) was included to control for possible differential availability of the HPV vaccine.

### Analytical strategy

Following the description of HPV vaccination prevalence and the prevalence of vaccine hesitancy, two multivariate logistic regression analyses were carried out. The first explored correlates of ever being vaccinated against HPV and the second correlates with vaccine hesitancy (defined as not wanting to be vaccinated). Independent variables in the former regression analysis were demographic (age and gender) and family characteristics (parents' education and religious upbringing), while the latter included participants' socio-demographic characteristics, knowledge about HPV, religiosity, and self-assessed STI risks. To adjust standard errors for clustered sampling design, multivariate analyses were carried out using the complex samples module in the IBM SPSS 27 statistical software package. Very few missing information (<3%) was observed in the dataset, which is probably due to the fact that participants were contractual members of a commercial online panel (i.e., used to filling out questionnaires).

## Results

Less than one-fifth (18.3%) of participants, 25.0% of women and 11.7% of men from the sample, reported ever being vaccinated against HPV. Almost two-thirds of vaccinated individuals (65.6%) were women. Approximately one in every five participants (21.9%) was unable to recall their HPV vaccination status. There was no substantial gender difference in this lack of recall (*p* = 0.263).

Willingness to get the vaccine was reported by slightly over a third of non-vaccinated participants (35.4%; 37.5% of women and 33.4% of men). Men were significantly more reluctant to vaccinate against HPV compared to women [$\chi_{\left(1\right)}^{2}$ = 12.02, *p* < 0.001, Cramer's *V* = 0.12].

The majority of sampled emerging adults (59.1%) knew about the link between HPV and cervical cancer. Of the rest, 36.1% reported that they did not know the answer. No significant gender difference in knowledge that HPV infection causes cervical cancer was observed (*p* = 0.612).

Next, the correlates of being vaccinated against HPV were assessed (Table 2). Participants who could not remember if they were ever vaccinated against HPV were excluded from the analyses. In the multivariate model, it was found that the odds of reporting being vaccinated against HPV were significantly higher in women compared to men [odds ratio (OR) = 2.46; 95% confidence interval (CI) 1.76–3.46; *p* < 0.001].

**Table 2:** Predictors and correlates of reporting ever being vaccinated against HPV (*n* = 751).

	**aOR**	**95% CI**
**Gender**		
Female	2.46^***^	1.76–3.46
**Age**	1.00	1.00–100
**Religious upbringing**	1.02	0.92–1.14
**Mother's education (college** = **reference)**		
Primary	2.03	0.98–4.20
Secondary	1.42	0.86–2.35
**Father's education (college** = **reference)**		
Primary	1.01	0.48–2.15
Secondary	0.75	0.48–1.17

aOR, odds ratio adjusted for the contribution of other independent variables; CI, confidence interval.

^***^p < 0.001.

Finally, we explored the correlates of the HPV vaccine hesitancy (Table 3). Only sexually active participants were included in the analysis. In the adjusted model, the odds of vaccine hesitancy (i.e., the individual does not want to get vaccinated) were lower among female participants (aOR = 0.60, *p* < 0.001), individuals who believed that they were at higher STI risk (aOR = 0.89, *p* = 0.010), and those who knew that HPV causes cervical cancer (aOR = 0.66, *p* = 0.005). In contrast, higher religiosity substantially increased the odds of reporting vaccine hesitancy (aOR = 1.16, *p* = 0.001).

**Table 3:** Predictors and correlates of HPV vaccine hesitancy, Croatia (*n* = 809).

	**aOR**	**95% CI**
**Gender**		
Female	0.60^***^	0.45–0.79
**Age**	1.03	0.93–1.13
**Education (years of formal education)**	0.93	0.86–1.01
**Socioeconomic standing**	1.20	0.95–1.51
**Religiosity (freq. of church attendance)**	1.16^**^	1.06–1.27
**Relationship status (married or cohabiting** = **reference)**		
In a relationship but not cohabiting	0.96	0.65–1.43
Single	0.85	0.53–1.38
**Settlement type (urban** = **reference)**		
Rural	0.86	0.62–1.19
**Self-assessed risk of STI**	0.89^*^	0.82–0.97
**HPV can result in cervical cancer**		
Yes	0.66^**^	0.48–0.88

aOR, odds ratio adjusted for the contribution of other independent variables; CI, confidence interval.

^*^p < 0.05, ^**^p < 0.01, ^***^p < 0.001.

## Discussion

The results of this study indicate low self-reported uptake of HPV vaccination among emerging adults in Croatia and a high level of HPV vaccine hesitancy. By 2017, several European countries achieved a full course HPV vaccine coverage of over 70% in female individuals in target age groups: Iceland (12–13), the United Kingdom (11–13), Norway (12–13), Spain (12), and Sweden (10–12) (2, 6). Some countries geographically close to Croatia with a similarly organized healthcare system achieved a substantially higher HPV-VCR in 2017, such as Slovenia (46% target age 11–12 years), Czech Republic (58% target age 13 years), and Italy (62% target age 11 years) (2, 6). The most common determinants of HPV vaccine hesitancy identified in European countries include the quality and quantity of available information about the HPV vaccine and its safety, and the lack of trust in health authorities (7). This indicates a large need for improvement of HPV-VCR in Croatia.

Low perceived risk of contracting an STI as one of the correlates of HPV vaccine hesitancy in our study could be explained by perceived feelings of invulnerability, which characterize the period of adolescence (8). The association that we found between religiosity and vaccine hesitancy has been well-explained in the literature and includes concerns that vaccination will promote sexual disinhibition and sex before marriage among emerging adults (9, 10).

A large proportion of participants in this survey did not know whether they had been vaccinated or not, which suggests a possible low perception and awareness of vaccination benefits. Low vaccination uptake overall among respondents and lower HPV vaccination status reported among men, as well as their higher reluctancy to HPV vaccination, were identified in our study, pointing out the need for targeted intervention, e.g., educational peer workshops. One of the cornerstones is the promotion of HPV vaccination awareness and uptake among adolescents and young adults and their parents (11, 12), as well as the implementation of male-specific HPV campaigns emphasizing the double benefit of HPV vaccination (individual protection and transmission prevention). Campaigns should also be aimed specifically at emerging adults, given that they are at an age when they still could be vaccinated through catch-up programs, and also because the information that they obtain about HPV prevention could have an impact on their decision to vaccinate their children in the future (13).

Our study highlighted the HPV knowledge gaps and indicates the need for more thorough health education and awareness-raising strategies regarding HPV transmission and the consequences of HPV infection, targeting a general population and in particular emerging adults. Increases in HPV-VCR can be achieved in Croatia by addressing these knowledge gaps and increasing awareness about HPV vaccine effectiveness and its availability through school-based interventions, as well as efficient and innovative vaccine delivery strategies.

School-based interventions should include the provision of educational materials about the efficacy and safety of the HPV vaccine for parents, children, and teachers. During primary and secondary education in Croatia, the health education of children is provided by biology teachers and school medicine practitioners. Education on sexual health, however, is very limited and often considered controversial. Since evidence shows that additional parental education on the efficacy of HPV can increase vaccine uptake in children, specialists in school and adolescent medicine should increase their efforts to educate parents about the benefits of vaccination (11). Improvements in vaccine delivery strategies should include the implementation of online tools that facilitate vaccination scheduling, according to the national vaccination program (for all students aged 10/11–14/15 years), and obtaining parental consent.

Apart from its strengths, the current study also has some limitations. Although our population-based sample is broadly representative of the national 18–25 years age cohort, the question of whether members of commercial panels, who routinely participate in various (incentivized) surveys, may be specific in some way remains relevant, e.g., professional respondents vs. altruistic ones, in that sense as well (14). In addition, the fact that we assessed vaccination against HPV in retrospect entailed the risk of memory bias. Finally, the fact that data collection took place during the COVID-19 pandemic may have affected participants' self-assessment of STI risk exposure.

## Conclusion

Given the potential public health ramifications of low HPV vaccination uptake, multidisciplinary approaches conducted in educational and healthcare settings should be undertaken to promote the benefits of the HPV vaccine for both women and men among parents and emerging adults, as well as to address misperceptions around vaccine side effects and effectiveness. HPV vaccination procedures with broader availability should be implemented. Further research is needed to elucidate the factors behind HPV vaccine hesitancy in Croatia, including associated social interaction, as that would additionally help in planning targeted interventions.

### Study implications

The current study provided the first population-level estimates of HPV vaccination coverage and HPV vaccine hesitancy in emerging Croatian adults. Its findings are of particular relevance for public health specialists and school and adolescent medicine practitioners who are responsible for vaccination against HPV. School settings should be used more effectively to raise awareness about the benefits of the HPV vaccine among students and their parents, as well as to scale up vaccination coverage in Croatia to reach the EU average.

## Data availability statement

The raw data supporting the conclusions of this article will be made available by the authors, without undue reservation.

## Ethics statement

The studies involving humans were approved by the Committee on Ethics Issues in Science and research of the Faculty of Humanities and Social Science, University of Zagreb, Zagreb, Croatia (Approval number 2019-14), and by the Ethical Committee of the Croatian Institute of Public Health, Zagreb, Croatia (Approval number 030-02/21-01/6−381-15-20-3). The studies were conducted in accordance with the local legislation and institutional requirements. The participants provided their written informed consent to participate in this study.

## Author contributions

TNB, IB, AŠ, and IAN contributed to the conceptualization and design of the manuscript. AŠ, TNB, IB, and MK contributed to the conceptualization and the methodology of the study and participated in research implementation and data collection. IB and AŠ participated in organizing the database, performed the statistical analysis, and results validation. TNB wrote the first draft of the manuscript. AŠ was responsible for investigation, data curation, and funding acquisition. All authors contributed to manuscript revision and editing, and have read and approved the submitted version.
