# Supplementary material for: Self-reported HPV vaccination status and HPV vaccine hesitancy in a nationally representative sample of emerging adults in Croatia
Source: Front Public Health. 2023 Nov 6;11:1182582. doi: 10.3389/fpubh.2023.1182582 (PMC10657860; doi:10.3389/fpubh.2023.1182582)
Supplement: Supplementary file 1 [file Table_1.docx]

Supplemental Table – Basic sociodemographic characteristics of the sample by gender (unweighted data; n=981)*

|  | Women | Men | Total |
| --- | --- | --- | --- |
|  | *n* (%) | *n* (%) | *n* (%) |
| Mother’s education  primary  secondary  tertiary  unknown | 56 (10.96)  346 (67.71)  106 (20.74)  3 (0.59) | 35 (7.45)  292 (62.13)  142 (30.21)  1 (0.21) | 91 (9.3)  638 (65.0)  248 (25.3)  4 (0.4) |
| Father’s education  primary  secondary  tertiary  unknown | 61 (11.94)  345 (67.52)  98 (19.18)  7 (1.37) | 35 (7.45)  296 (62.98)  137 (29.15)  2 (0.43) | 96 (9.8)  641 (65.3)  235 (243.0)  9 (0.9) |
| Relationship status  married  cohabiting  in a relationship but not cohabiting  single  something else | 25 (4.89)  96 (18.78)  239 (46.77)  149 (29.16)  2 (0.39) | 13 (2.77)  58 (12.34)  182 (38.72)  214 (45.53)  3 (0.64) | 38 (3.9)  154 (15.7)  421 (42.9)  363 (37.0)  5 (0.5) |
| Settlement type  rural  urban | 222 (43.44)  289 (56.56) | 144 (30.64)  326 (69.36) | 366 (37.3)  615 (62.7) |
|  | *M* (*SD*) | *M* (*SD*) | *M* (*SD*) |
| Age (years) | 22.6 (1.93) | 22.1 (2.16) | 22.3 (2.06) |
| Education (years of formal education) | 7.8 (2.08) | 6.9 (2.21) | 7.4 (2.19) |
| Socioeconomic standing (5 -point Likert scale)** | 3.1 (0.67) | 3.3 (0.68) | 3.2 (0.68) |
| Religious upbringing (7-point scale)** | 4.7 (1.61) | 4.5 (1.62) | 4.6 (1.61) |
| Religiosity (freq. of church attendance)** | 3.7 (1.53) | 3.5 (1.55) | 3.6 (1.54) |

*Numbers do not always add up due to rounding up

** Explained under the section Measures
